# Supplementary material for: Field emission effect in triboelectric nanogenerators
Source: Nat Commun. 2025 May 20;16:4706. doi: 10.1038/s41467-025-59927-4 (PMC12092747; doi:10.1038/s41467-025-59927-4)
Supplement: Supplementary file 1 — Supplementary Information [file 41467_2025_59927_MOESM1_ESM.pdf]

Supplementary information for

Field emission effect in triboelectric nanogenerators

Di Liu[1,2,δ], Yikui Gao[1,3,δ], Wenyan Qiao[1,3], Linglin Zhou[1,3,4], Lixia He[1,3], Cuiying Ye[1,3], Bingzhe Jin[1,3], Baofeng Zhang[5], Zhong Lin Wang[1,3,6,*], Jie Wang[1,3,4,*]

[1]Beijing Key Laboratory of Micro-Nano Energy and Sensor, Center for High-Entropy Energy and Systems, Beijing Institute of Nanoenergy and Nanosystems, Chinese Academy of Sciences, Beijing 101400, P. R. China

[2]Department of Mechanical Engineering, The Hong Kong Polytechnic University, Hong Kong 999077, P. R. China

[3]College of Nanoscience and Technology, University of Chinese Academy of Sciences, Beijing 100049, P. R. China

[4]Guangzhou Institute of Blue Energy, Knowledge City, Huangpu District, Guangzhou 510555, P. R. China

[5]Hubei Key Laboratory of Automotive Power Train and Electronic Control, School of Automotive Engineering, Hubei University of Automotive Technology, Shiyan, 442002, P. R. China

[6]Yonsei Frontier Lab, Yonsei University, Seoul 03722, Republic of Korea

[δ]D. Liu and Y. Gao contributed equally to this work.

*Corresponding Authors: Z. L. Wang: zhong.wang@mse.gatech.edu;
J. Wang: wangjie@binn.cas.cn

This file includes:

Supplementary Figure 1-14

Supplementary Note 1-10

References

Supplementary Figures

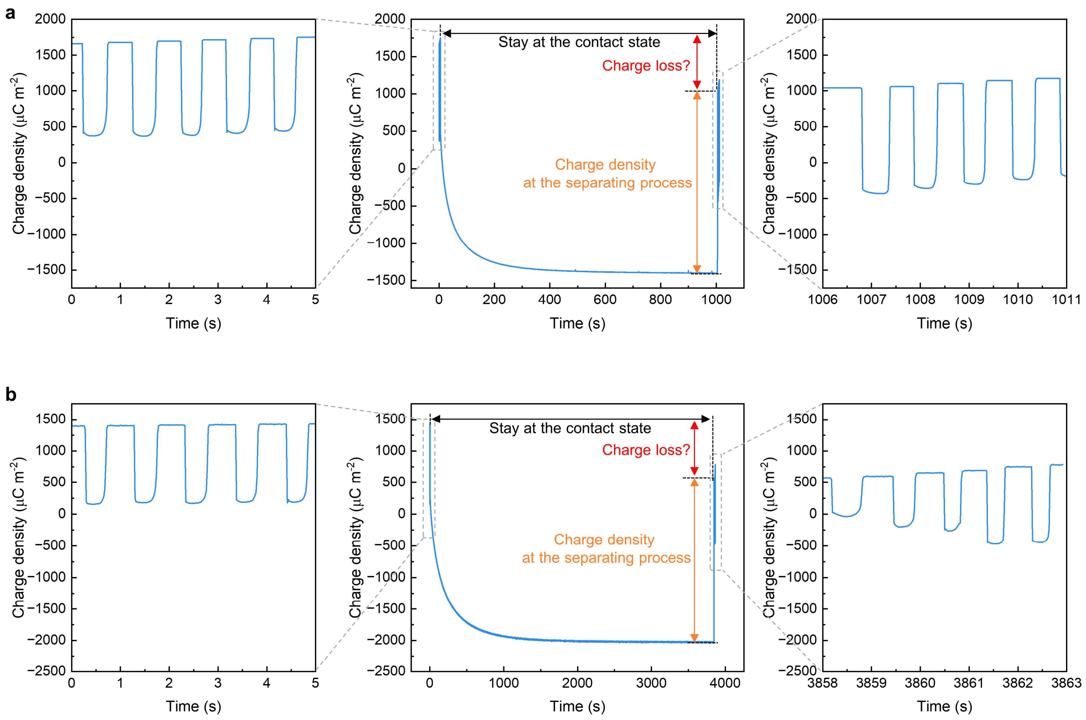

Supplementary Fig. 1. The output charge density of polyimide (6 μm) and copper under vacuum conditions. a and **b** Output charge density with different contact time. The charge dissipation still exists even when the contact time is extended to surpass an hour. Source data are provided as a Source data file.

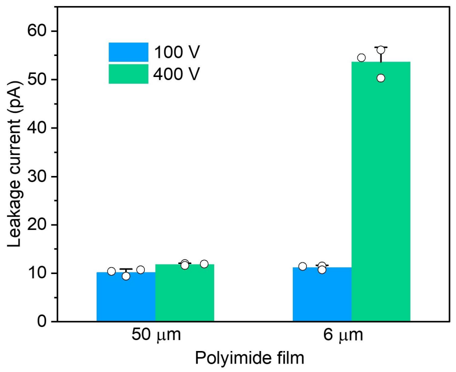

Supplementary Fig. 2. The leakage current of polyimide film. With the film thickness decreasing and the applied voltage increasing, the leakage current is greatly increased. Data are presented as mean values ± SD, $n = 3$ independent measurements. Source data are provided as a Source data file.

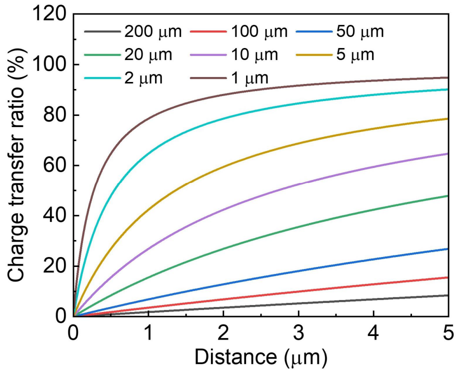

Supplementary Fig. 3. Charge transfer ratio of CS-TENG with different dielectric thickness when the separating distance is confined to within 5 μm. The relative permittivity of the polyimide is 3.67 according to **Supplementary Fig. 4**. Source data are provided as a Source data file.

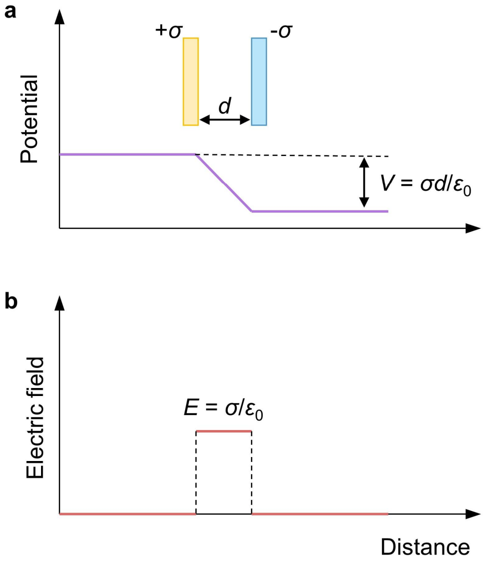

Supplementary Fig. 4. The potential and electric field distribution between two charged plates. a Potential and **b** electric field in a simplified model. Theoretically, the potential is described as $V=\sigma d/\varepsilon_0$ and the electric field is described as $E=\sigma/\varepsilon_0$ (σ is SCD and d is the gap distance between the two plates.).

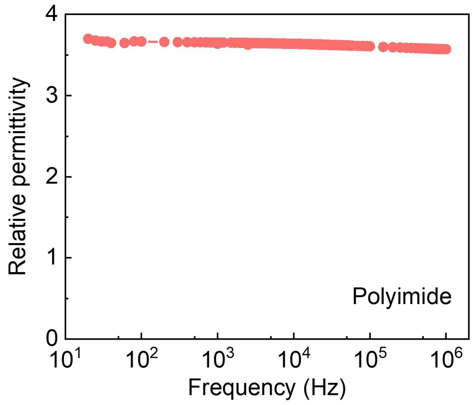

Supplementary Fig. 5. The relative permittivity of the polyimide. (3.67, 100 Hz). Source data are provided as a Source data file.

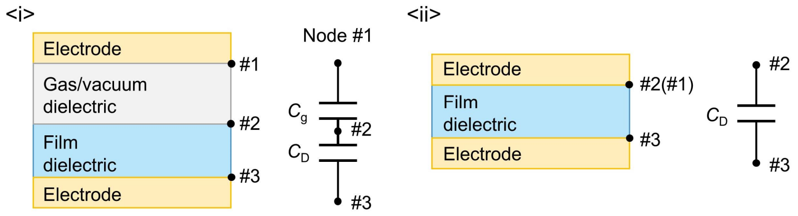

Supplementary Fig. 6. The equivalent electric circuit of CS-TENG <i> at the separate state and <ii> at the contact state. The equivalent circuit model of the CS-TENG in the contact state is a film dielectric capacitor (C_D), while it is C_D and the gas dielectric capacitor (C_g) connected in series in the separated state. Obviously, C_g is much smaller than C_D, so voltage in the separated state (V_{OC}) is different from the voltage in the contact state (V'_{OC}) theoretically.

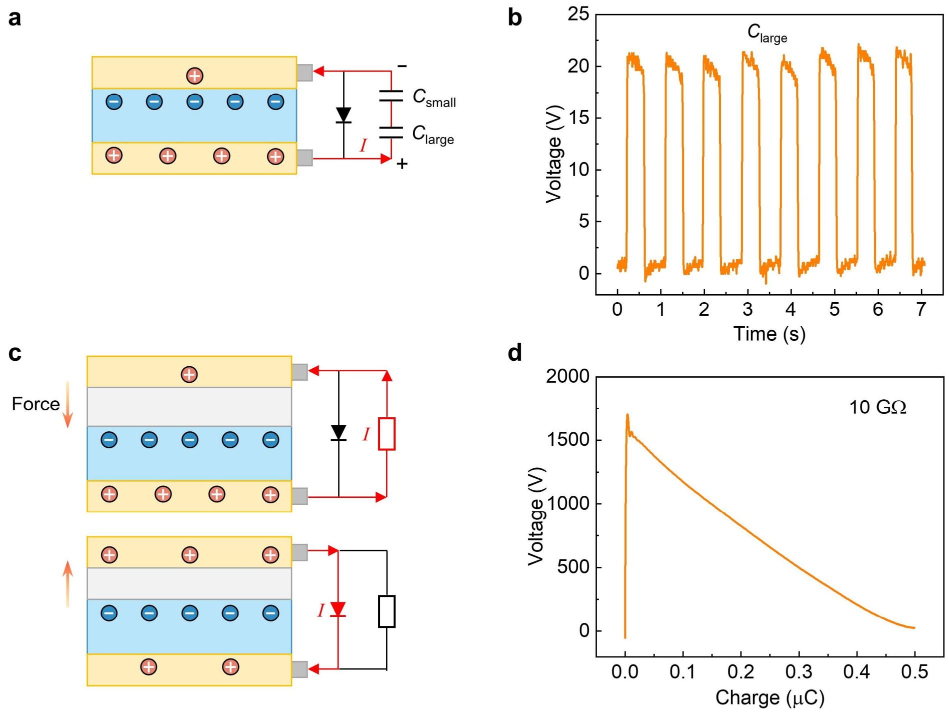

Supplementary Fig. 7. Output performance of CS-TENG with the large parasitic capacitance from separate to contact. a Schematic diagram shows voltage measurement of CS-TENG at the contact state by charging capacitors. **b** Voltage of the large capacitor. **c** Schematic diagram shows voltage measurement of CS-TENG at the contact state by monitoring the current of external load resistance. **d** The *V*-*Q* curve of CS-TENG at contact state under the load of 10 GΩ. Source data are provided as a Source data file.

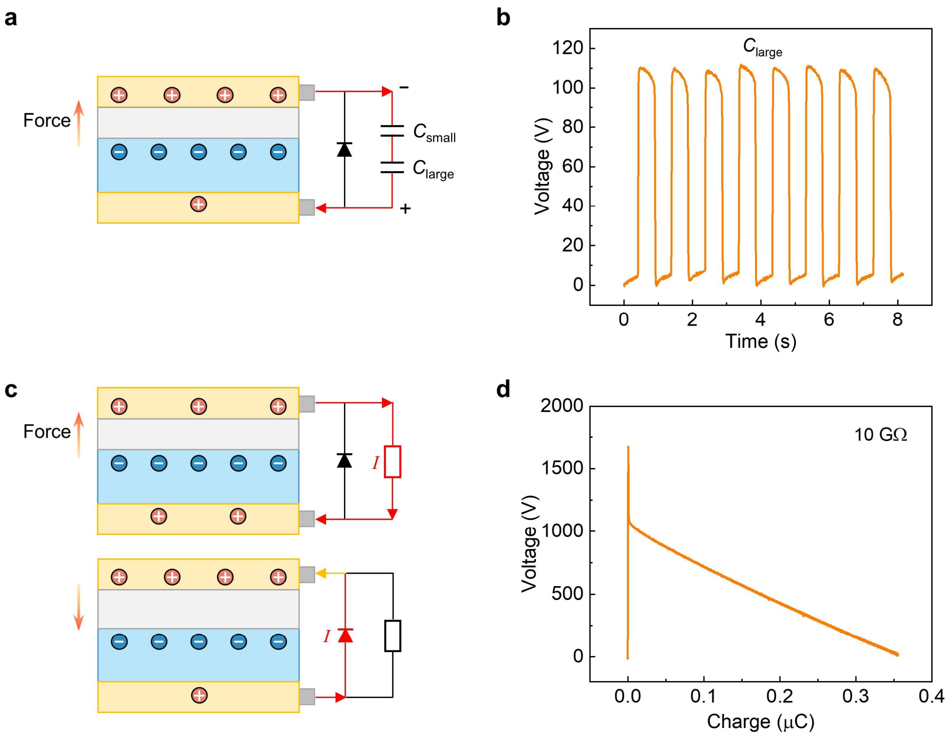

Supplementary Fig. 8. Output performance of CS-TENG at the separate state, without reducing the parasitic capacitor. a Schematic diagram shows voltage measurement of CS-TENG at the separate state by charging capacitors. **b** Voltage of the large capacitor. **c** Schematic diagram shows voltage measurement of CS-TENG at the separate state by monitoring the current of external load resistance. **d** The *V*-*Q* curve of CS-TENG at separate state under the load of 10 GΩ. Source data are provided as a Source data file.

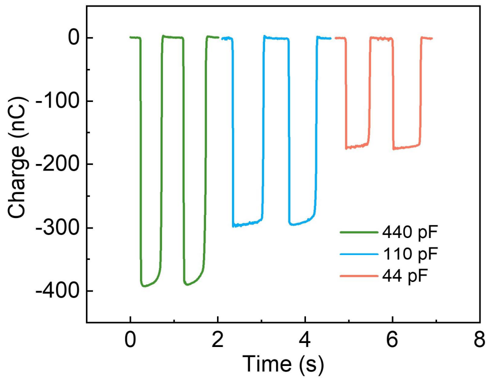

Supplementary Fig. 9. The output charge curve of CS-TENG for charging different capacitors at contact process. Source data are provided as a Source data file.

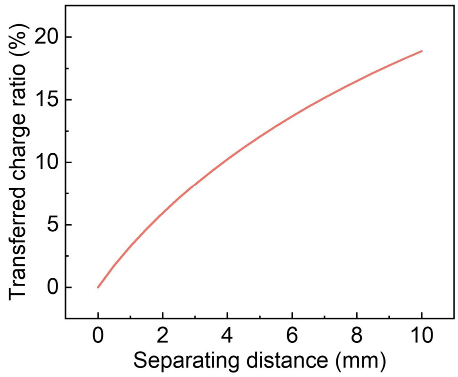

Supplementary Fig. 10. Simulated transferred charge ratio with separating distance of S-TENG. Source data are provided as a Source data file.

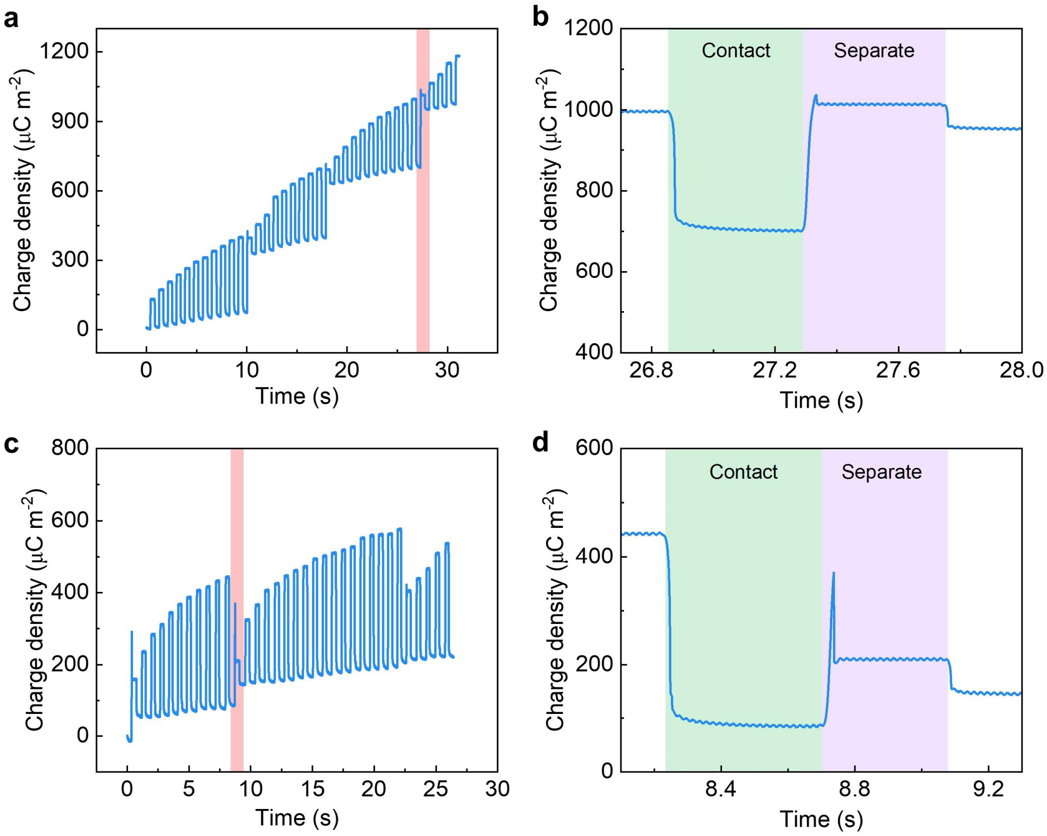

Supplementary Fig. 11. Short-circuit transferred charge of S-TENG of different materials under vacuum conditions. a PVC. **b** The enlarged figure at figure a. **c** FEP. **d** The enlarged figure at figure c. Source data are provided as a Source data file.

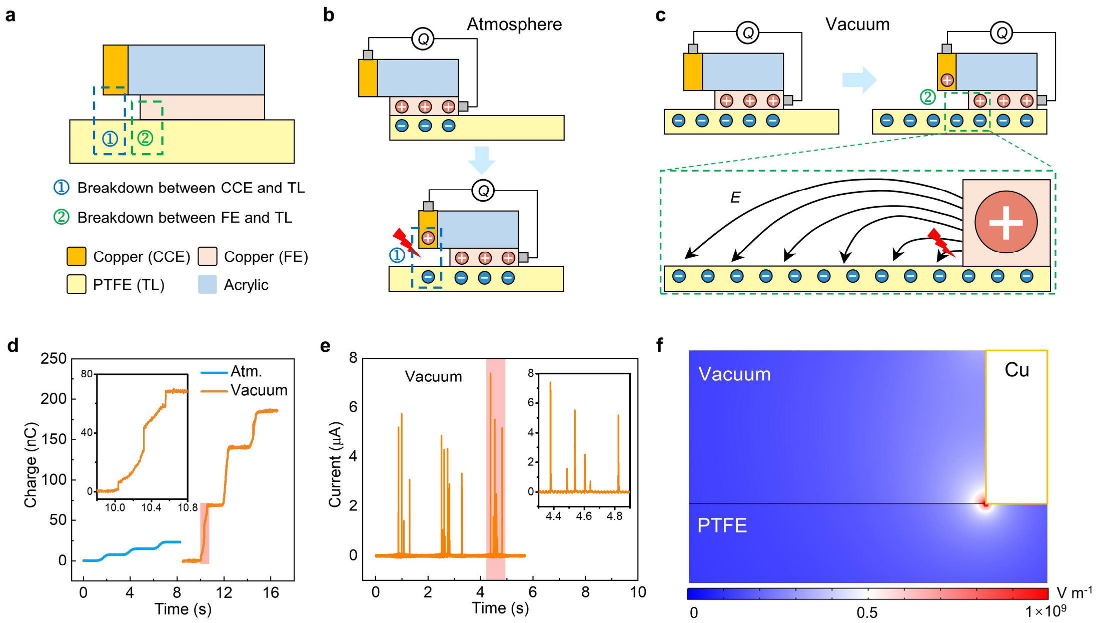

Supplementary Fig. 12. Field emission in sliding mode TENG. a Two breakdown domains in DC-TENG. ① is the breakdown phenomenon between CCE and TL; ② is the breakdown phenomenon between FE and TL. **b** Working mechanism of DC-TENG in atmosphere condition arising from air breakdown. **c** Working mechanism of DC-TENG in vacuum condition arising from field emission. The enlarged figure shows the schematic electric field intensified at the corner of FE. **d** Output charge curves of DC-TENG in atmosphere and vacuum conditions. The enlarged figure shows the separated steps of charge growth in vacuum condition. **e** Output current curve of DC-TENG in vacuum condition. The enlarged figure shows the separated current peak of DC-TENG. **f** The simulated electric field distribution in a DC-TENG. The electric field at the corner around the left of FE is easy beyond 10^9 V m^{-1}. The parameters in the DC-TENG for simulation are as follows: the relative dielectric constant of film dielectric is 2.5; the surface charge density of film dielectric is 500 μC m^{-2}. Source data are provided as a Source data file.

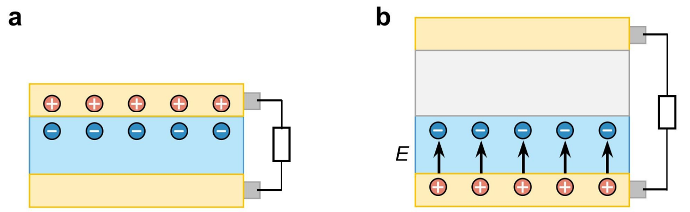

Supplementary Fig. 13. Charge distribution in CS-TENG at different states. a Contact state. **b** Separate state. The schematic diagram indicates that dielectric breakdown is most likely to occur when the CS-TENG are sufficiently separated.

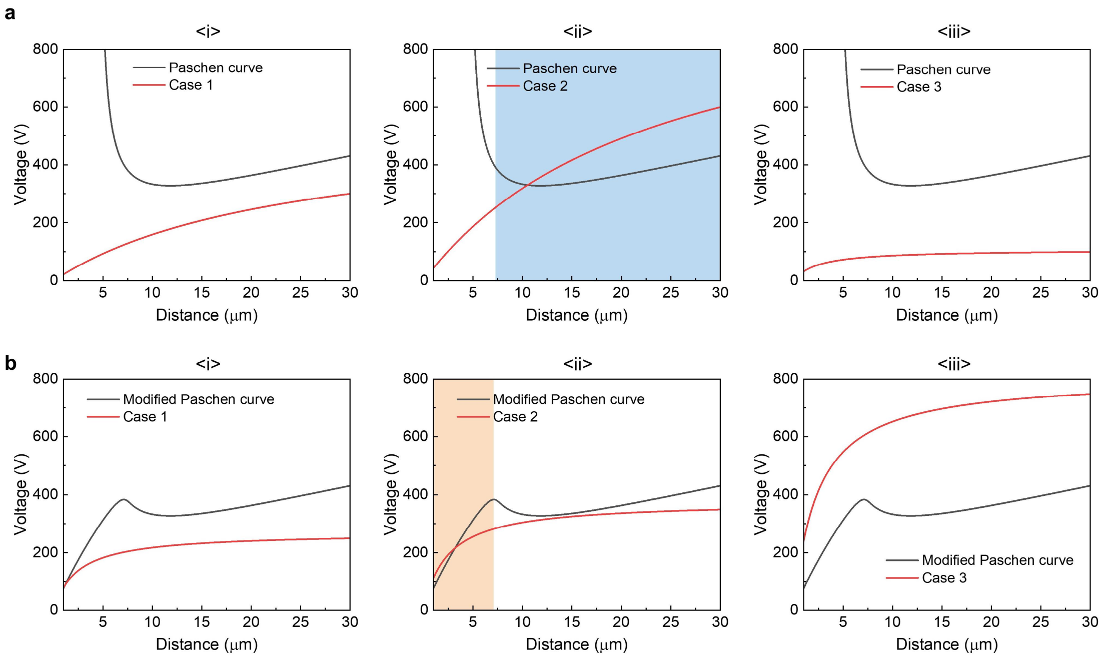

Supplementary Fig. 14. Relationships between the gap voltage in CS-TENG and the breakdown voltage. a Air gap voltage in CS-TENG and the breakdown voltage described by the Paschen's curve. <i> σ_D=200 μC m^{-2}; d_D=50 μm; $\varepsilon_D/\varepsilon_0$=2.1. <ii> σ_D=400 μC m^{-2}; d_D=50 μm; $\varepsilon_D/\varepsilon_0$=2.1. <iii> σ_D=400 μC m^{-2}; d_D=5 μm; $\varepsilon_D/\varepsilon_0$=2.1. **b** Air gap voltage in CS-TENG and the breakdown voltage described by the modified Paschen's curve. <i> σ_D=1000 μC m^{-2}; d_D=5 μm; $\varepsilon_D/\varepsilon_0$=2.1. <ii> σ_D=1400 μC m^{-2}; d_D=5 μm; $\varepsilon_D/\varepsilon_0$=2.1. <iii> σ_D=3000 μC m^{-2}; d_D=5 μm; $\varepsilon_D/\varepsilon_0$=2.1. Source data are provided as a Source data file.

Supplementary Notes

Supplementary Note 1. Breakdown theory.

The breakdown mechanism of gas is usually based on Townsend avalanche, which is described by the empirical relationship between breakdown voltage (V_b) and the product of gap distance (d_g) and gas pressure (p). Generally, the breakdown criterion is derived based on two important parameters: the electron impact ionization coefficient α and the secondary electron emission coefficient γ.

$$\gamma\left(e^{\alpha d_g} - 1\right) = 1 \qquad (1)$$

where α describes the generation of ions by electron impact and the corresponding equation is as follows.

$$\alpha = Ape^{-Bpd_g/V_b} \qquad (2)$$

where A, B are two constants relating to gas compositions, and p is the gas pressure. Thus, the following equation can be obtained.

$$V_b = \frac{Bpd_g}{\ln(Apd_g) + \ln\left[\frac{1}{\ln\left(\frac{1}{\gamma}+1\right)}\right]} \qquad (3)$$

This is the traditional Paschen curve.

For the breakdown voltage in micro-discharges, the following equation is given:[1,2]

$$V_b = \frac{d_g(D+Bp)}{\ln(Apd_gK)} \qquad (4)$$

where K, D are cathode material-dependent and gas-dependent constants, described by the following equation.

$$D = (6.85 \times 10^7)\frac{\Phi^{3/2}}{\beta} \qquad (5)$$

Here, β is a field enhancement factor and Φ is the work function of the cathode material. It is noted that the breakdown voltage in equation (4) is a function of p and d_g separately rather than a function of pd_g.

In addition, the modified Paschen curve is described as the following equation:

$$\left(\gamma + Ke^{-Dd_g/V_b}\right)\left[e^{Apd_g\exp(-Bpd_g/V_b)} - 1\right] = 1 \qquad (6)$$

In this paper, the parameters are chosen as: α, 0.008136; K, 10^7; p, 101.25 kPa; A, 10.95 $(\text{m}\times\text{Pa})^{-1}$; B, 273.8 V/ (m×Pa); Φ, 4.36 eV (for polyimide); β, 50. Therefore, the

corresponding curves in **Fig. 2c** can be obtained. The curves in **Fig. 2d** are obtained by the ratio of voltage to gap distance (the red line) from the modified Paschen curve in **Fig. 2c**.

Supplementary Note 2. SCD improvement in CS-TENG.

Given that the output power of TENG is proportional to the square of the SCD and the sensor sensitivity is directly proportion to the SCD, it is vital to enhance the SCD of TENG to improve its output performance. In the last decade, the SCD of TENG has been improved from about 50 μC m^{-2} to 1.25 mC m^{-2} by optimizing the triboelectric performance and suppressing the air breakdown effect, including materials choice, surface modification (physical and chemical modification), soft and fragmental contact (improve contact intimacy), structural optimization, ultrathin dielectric layer, environmental control (high atmosphere pressure condition, breakdown resistant gas condition, high vacuum condition), and so on.[3] In addition, the charge pump technology was also proposed to break through the charge limit from triboelectrification. Combinging with the thin dielectric layer to restrict air breakdown, the effective charge density in the TENG based on the charge pump technology can be boosted to around several mC m^{-2}. It is no doubt that the effective charge density would be further increased by choosing the thinner dielectric layer or optimizing the relative permittivity of dielectric layer as well as decreasing the leakage current in the dielectric layer. It is worth noting that the high effective charge density is achieved in short-circuit condition where electrons can freely flow in external circuit, so the inner electric field in TENG is restricted to some extent. However, for power generation, TENG in short-circuit condition produces no power in the external circuit. In other words, TENG must connect with the load for power generation. At the load condition, the enhanced electric field in TENG greatly reduces the permitted SCD, and that's why the output power or energy is generally less than the claculated power or energy based on the short-circuit charges.

Supplementary Note 3. Effects of parasitic capacitance on the performance of output voltage and energy of TENG.

Given that the little inherent capacitance of TENG, the parasitic capacitance generally has significant impacts on the output voltage and energy of TENG, especially for high-voltage power sources and high-power density applications.[4,5] To achieve a high output voltage, the common methods rely on increasing SCD, decreasing the inherent capacitance within TENG, and increasing the device area. The core issue is optimizing the TENG's inherent capacitor as well as minimizing the impacts of parasitic capacitance simultaneously. The parasitic capacitor is commonly connected with the inherent capacitance of TENG in parallel. Previous works have demonstrated that the output voltage of TENG cannot be continuously increased by decreasing the inherent capacitance within the TENG. Because the reduced inherent capacitance amplifies the effects of parasitic capacitance on the output voltage of TENG. For a manufactured device, shorten the length of the conductive wire, away from the conductors and charged body, and effective electromagnetic shielding are effective methods to reduce the impacts of parasitic capacitance on the output voltage of TENG.

At the normal condition, we found that the measured output voltage of the CS-TENG at the contact state, whether by the method of series capacitance voltage vision (C_{large}=22 nF, C_{small}=220 pF, **Supplementary Fig. 7a** and **b**) or by the Ohm's law (1703 V, **Supplementary Fig. 7c** and **d**), is smaller than the theoretical value (2140 V), calculated by the following equation:

$$V = \frac{Qd}{\varepsilon_0 \varepsilon_r S} \tag{7}$$

where Q is the output charges (499 nC); d is the thickness of dielectric layer (50 μm); ε_0 is the vacuum dielectric permittivity; ε_r is the relative permittivity of the dielectric layer (3.67 for polyimide, **Supplementary Fig. 5**); S is the effective triboelectrification area (4 cm^2). However, the output charge maintains at around 500 nC (**Supplementary Fig. 7d**).

Given that the inherent capacitance of CS-TENG at separate state is much smaller than the inherent capacitance of CS-TENG at contact state (0.233 nF), the output

voltage of the CS-TENG at separate state should be very high, but meantime, it is also significantly restricted by the parasitic capacitor. Despite the output voltage measured by the series capacitor voltage vision method can be up to around 10 kV (**Supplementary Fig. 8a** and **b,** the voltage error of series capacitance calculation is large because the ratio of capacitance is 100, which also magnifies the error.), but the measured voltage by the Ohm's law is lower than 2 kV (**Supplementary Fig. 8c** and **d**). The two measured voltage values are much smaller than the theoretical voltage. Moreover, the output charge decreases from 500 nC to around 350 nC, indicating the charge loss in vacuum condition.

In summary, the output voltage of TENG is significantly influenced by the parasitic capacitance, so we reduced the parasitic capacitance as much as possible for the following data acquirement. Thus, the output voltage of TENG can be boosted and the field emission is also amplified.

Supplementary Note 4. Output energy and voltage of CS-TENG at large external loads from contact to separate.

As we reduced the parasitic capacitance as much as possible, we found that the output charges of CS-TENG from contact to separate gradually decrease with the external load resistance. However, the output voltage keeps nearly stable at around 10 kV rather than decreased with the tendency of output charge (**Fig. 3f**). This is very interesting and puts us forward to think out the reasons. Comparing with the short-circuit condition, there is an additional voltage across the gap at the load conditions.[5] With the increasing of load resistance, the additional voltage to the gap also increases, leading to the surface charge loss from breakdown occurred at a smaller gap distance, so the induced transferred charges in external circuit will decrease. We can find that the maximum voltage under various load resistances in the *V-Q* curves keeps stable at around 10 kV, which is independently with the transferred charges, implying a limiting factor for the gap voltage, so the output energy is also limited.

Supplementary Note 5. Measurement of maximized output energy cycle of CS-TENG with the external load method.

Ideally, the maximized output energy cycle of CS-TENG is achieved at the condition that the voltage is built at open-circuit condition and the charge is obtained at short-circuit condition. Generally, the maximized output energy cycle of CS-TENG can be obtained by plotting the output charge and voltage across the external load resistance, if the external load resistance is large enough. However, the output performance of CS-TENG is restricted by air breakdown as demonstrated by many previous works, so the output energy cycle of CS-TENG is also affected by air breakdown.[6] In vacuum condition, the air breakdown effect is fully removed, and the maximized output energy cycle of CS-TENG should be obtained. In the previous work, we found that the output energy of CS-TENG is highly dependent on the parasitic capacitance, because the output voltage is greatly reduced with the large parasitic capacitance. Here, by reducing the parasitic capacitance as much as possible, we found the maximized output energy cycle of CS-TENG collapsed even in vacuum condition. This result indicates that the maximized output energy cycle of CS-TENG cannot be directly obtained in this method.

Supplementary Note 6. Measurement of maximized output energy cycle of CS-TENG with the external capacitor method.

The maximized output energy cycle of CS-TENG can also be obtained by plotting the voltage and stored charges of various capacitors in the *V*-*Q* figure, in which various capacitors are charged by the CS-TENG.[6] As shown in **Fig. 3g-i**, if a capacitor is connected with the CS-TENG, the charges stored in the inherent capacitor of CS-TENG will flow into the external capacitor, i.e., charging the external capacitor. For a fixed external capacitor, there will a group of voltage and charge (V_1 and Q_1). And so on, several groups of voltage and charge (V_2 and Q_2, V_3 and Q_3, …, V_n and Q_n) can be obtained by changing the external capacitor. By plotting these values in a *V*-*Q* figure and linearly connecting these points, the intercept between the horizontal and vertical axes should represent the maximized output charge and voltage of CS-TENG, respectively, and the reciprocal of slop of this curve should represent the inherent capacitor of the CS-TENG. Thus, the maximized output energy cycle of CS-TENG would be obtained by this method, theoretically.

Supplementary Note 7. The limiting factor implied by a smaller C_f.

As shown in **Fig. 3g**, the CS-TENG is used for charging different C_f, in which a diode is parallel connected to realize the charge reset in the CS-TENG. It is clearly that the charging curves for the small C_f (**Fig. 3k**) are different from the charging curve for a large C_f (**Fig. 3j**). With the CS-TENG from contact to separate, the output charge gradually increases and then follows a sharp decline, rather than the common increased charging curve. Specifically, the maximized output charge value gradually decreases with the decreasing of the C_f. If we calculated the voltage of these capacitors, we can find that the voltage of these capacitors keeps nearly stable. Because, to reach a same voltage value for different capacitors, the required charge quantity is smaller for a small capacitor. The results in **Fig. 3l** demonstrate that the output voltage is approximately several kV. Despite that the smaller capacitor would be charged to a higher voltage value, but the experimental results were different, implying a limiting factor for output voltage again.

Supplementary Note 8. The represented values of output energy density of CS-TENG.

The energy of TENG is stored in film dielectric layer and gas/vacuum dielectric. Therefore, we divide previous research about TENG's energy into these two categories for comparison.

The first category, energy is stored in the gas/vacuum dielectric. In 2020, Liu et al. used a CS-TENG with an area of 20 cm^2 to achieve an output energy density of 56 mJ m^{-2} (The charge density is 83 μC m^{-2} and the voltage is 1300 V.).[7] Due to the significant influence of parasitic capacitance, the energy density is low. In 2022 and 2021, Wang et al. used the CS-TENG with an area of 100 cm^2 to decrease the influence of parasitic capacitance, and the output energy density of 142 mJ m^{-2} and 108 mJ m^{-2} are achieved (The charge density is 42 μC m^{-2} and 45 μC m^{-2} respectively, and the voltage is 7500 V and 6000 V respectively.).[8,9]

The second category, energy is stored in the film dielectric layer. In 2023, Gao et al. reported a strategy for suppressing electrostatic breakdown and decreasing the influence of parasitic capacitance in CS-TENG, an output energy density of 130 mJ m^{-2} is achieved (The charge density is 250 μC m^{-2} and the voltage is 1020 V.) in CS-TENG with the area of 10 cm^{-2}.[5] In 2023, Zhang et al. reported a three-dimensional fractal structured nanogenerator (FSNG), which can efficiently collect the triboelectrification induced space-distributed Maxwell's displacement current, and an output energy density of 389 mJ m^{-2} is achieved (The charge density is 250 μC m^{-2} and the voltage is 1020 V.) in sliding-mode TENG with the area of 16 cm^{-2}.[10] In 2023, Wu et al. leverage the opposite-charge-enhancement effect and the transistor-like device design, achieving an output energy density of 395 mJ m^{-2} in sliding-mode TENG with the area of 25 cm^{-2}. (the charge density is 400 μC m^{-2} and the voltage is 2520 V).[11]

Supplementary Note 9. Field emission effect in sliding mode TENGs.

For the sliding mode TENGs, including free-standing mode TENG, lateral sliding mode TENG, and direct-current TENG (DC-TENG), the most likely location for breakdown is the corner between the slider and the stator for these working modes because of the intensified electric field.[6] To clearly illustrate the field emission effect in sliding mode TENGs, we take the DC-TENG as an example. The working mechanism of DC-TENG is based on triboelectrification and electrostatic breakdown in atmosphere condition. In principle, the DC-TENG should have no output in vacuum condition where the air breakdown effect is fully removed, as the description in our previous work. However, we find that a large external force, higher than the previous work, can produce more triboelectric charges on the surface of dielectric layer, so the intensified electric field around the corner of the FE in DC-TENG can breakdown the gap even in vacuum conditions. Different from the utilization of air breakdown between the charge collection electrode (CCE) and the triboelectric layer (TL) in atmosphere conditions (① in **Supplementary Fig. 12a** and **b**), DC-TENG utilizes field emission between the frictional electrode (FE) and TL in vacuum conditions (② in **Supplementary Fig. 12a** and **c**). This breakdown will result in the fast current flow in external circuit with high peak values but short-duration pulse (**Supplementary Fig. 12d** and **e**), which is different from the constant current output of DC-TENG in atmospheric conditions. Therefore, the working mechanism of DC-TENG in vacuum condition is based on triboelectrification and electrostatic breakdown that occurred at around FE, rather than the previous breakdown domain around CCE. The COMSOL simulation result also suggests the possibility of occurrence of field emission, leading to charge release from FE to TL (**Supplementary Fig. 12c**). It is noted that field emission around the corner of FE could happen only when surface charge density is very high.

Supplementary Note 10. The limitations for charge excitation TENGs.

Generally, if only the Townsend avalanche is considered in the breakdown model of charge excitation TENGs, there are three cases for the relationships between the gap voltage in the charge excitation TENGs and the breakdown voltage described by the Paschen's curve. For the low effective charge density, there is no breakdown effect (case 1 in **Supplementary Fig. 14a** <i>). As the effective charge density increases, the breakdown effect occurs (case 2 in **Supplementary Fig. 14a** <ii>). By decreasing the dielectric thickness or increasing the permittivity of dielectric film, the breakdown effect caused by Townsend avalanche can be avoided, so a high effective charge density can be maintained (case 3 in **Supplementary Fig. 14a** <iii>). That's why a very high effective charge density can be achieved in the charge excitation TENGs by using the dielectric film with the thin thickness but high permittivity. Theoretically, the critical charge density for Townsend avalanche is very high. However, in experiment, the high charge density has not been achieved. The traditional view is that this is attributed to leakage current.

Here, we utilize the modified Paschen's curve to substitute the initial Paschen's curve, with the consideration of field emission. We can find that the gap voltage in the charge excitation TENG gradually increases with the charge density increasing (case 1 in **Supplementary Fig. 14b** <i>). Then, the air gap voltage will exceed the breakdown voltage within a few micrometers, but it is not exceeding the breakdown voltage at the gap of tens micrometers described by Townsend avalanche (case 2 in **Supplementary Fig. 14b** <ii>). As the charge density increases again, the air gap voltage will exceed the breakdown voltage described by both field emission and Townsend avalanche (case 3 in **Supplementary Fig. 14b** <iii>). These results indicate that the micro breakdown could be another limitation factor for charge excitation TENGs. Because it is to be occurred prior to air breakdown in some cases.

References

1. Go D. B. & Venkattraman A. Microscale gas breakdown: Ion-enhanced field emission and the modified Paschen's curve. *J Phys. D Appl. Phys.* **47**, 503001 (2014).
2. Loveless A. M. & Garner A. L. Scaling laws for gas breakdown for nanoscale to microscale gaps at atmospheric pressure. *Appl. Phys. Lett.* **108**, 234103 (2016).
3. Liu D. *et al.* Standardized measurement of dielectric materials' intrinsic triboelectric charge density through the suppression of air breakdown. *Nat. Commun.* **13**, 6019 (2022).
4. Dai K. *et al.* Simulation and structure optimization of triboelectric nanogenerators considering the effects of parasitic capacitance. *Nano Res.* **10**, 157-171 (2017).
5. Gao Y. *et al.* Achieving high-efficiency triboelectric nanogenerators by suppressing the electrostatic breakdown effect. *Energy Environ. Sci.* **16**, 2304-2315 (2023).
6. Gao Y. *et al.* Spontaneously established reverse electric field to enhance the performance of triboelectric nanogenerators via improving Coulombic efficiency. *Nat. Commun.* **15**, 4167 (2024).
7. Liu W. *et al.* Switched-capacitor-convertors based on fractal design for output power management of triboelectric nanogenerator. *Nat. Commun.* **11**, 1883 (2020).
8. Wang Z. *et al.* Ultrahigh electricity generation from low-frequency mechanical energy by efficient energy management. *Joule* **5**, 441-455 (2021).
9. Wang Z*, et al.* Giant performance improvement of triboelectric nanogenerator systems achieved by matched inductor design. *Energy Environ. Sci.* **14**, 6627-6637 (2021).
10. Zhang L. *et al.* Collecting the space-distributed Maxwell's displacement current for ultrahigh electrical density of TENG through a 3D fractal structure design. *Energy Environ. Sci.* **16**, 3781-3791 (2023).
11. Wu H., Wang S., Wang Z. & Zi Y. Achieving ultrahigh instantaneous power density of 10 MW/m by leveraging the opposite-charge-enhanced transistor-like triboelectric nanogenerator (OCT-TENG). *Nat. Commun.* **12**, 5470 (2021).
